# Complete genome sequence of *Nocardiopsis dassonvillei* type strain (IMRU 509^T^)

**DOI:** 10.4056/sigs.1363462

**Published:** 2010-11-30

**Authors:** Hui Sun, Alla Lapidus, Matt Nolan, Susan Lucas, Tijana Glavina Del Rio, Hope Tice, Jan-Fang Cheng, Roxane Tapia, Cliff Han, Lynne Goodwin, Sam Pitluck, Ioanna Pagani, Natalia Ivanova, Konstantinos Mavromatis, Natalia Mikhailova, Amrita Pati, Amy Chen, Krishna Palaniappan, Miriam Land, Loren Hauser, Yun-Juan Chang, Cynthia D. Jeffries, Olivier Duplex Ngatchou Djao, Manfred Rohde, Johannes Sikorski, Markus Göker, Tanja Woyke, James Bristow, Jonathan A. Eisen, Victor Markowitz, Philip Hugenholtz, Nikos C. Kyrpides, Hans-Peter Klenk

**Affiliations:** 1 DOE Joint Genome Institute, Walnut Creek, California, USA; 2 Los Alamos National Laboratory, Bioscience Division, Los Alamos, New Mexico, USA; 3 Biological Data Management and Technology Center, Lawrence Berkeley National Laboratory, Berkeley, California, USA; 4 Oak Ridge National Laboratory, Oak Ridge, Tennessee, USA; 5 HZI – Helmholtz Centre for Infection Research, Braunschweig, Germany; 6 DSMZ - German Collection of Microorganisms and Cell Cultures GmbH, Braunschweig, Germany; 7 University of California Davis Genome Center, Davis, California, USA

**Keywords:** Gram-positive, aerobic, pathogen, mesophile, non alkaliphilic, zig-zag-shaped mycelium, actinomycetoma, conjunctivitis, cholangitis, *Nocardiopsaceae*, GEBA

## Abstract

*Nocardiopsis dassonvillei* (Brocq-Rousseau 1904) Meyer 1976 is the type species of the genus *Nocardiopsis*, which in turn is the type genus of the family *Nocardiopsaceae*. This species is of interest because of its ecological versatility. Members of *N. dassonvillei* have been isolated from a large variety of natural habitats such as soil and marine sediments, from different plant and animal materials as well as from human patients. Moreover, representatives of the genus *Nocardiopsis* participate actively in biopolymer degradation. This is the first complete genome sequence in the family *Nocardiopsaceae*. Here we describe the features of this organism, together with the complete genome sequence and annotation. The 6,543,312 bp long genome consist of a 5.77 Mbp chromosome and a 0.78 Mbp plasmid and with its 5,570 protein-coding and 77 RNA genes is a part of the *** G****enomic* *** E****ncyclopedia of* *** B****acteria and* *** A****rchaea * project.

## Introduction

Strain IMRU 509^T^ (= DSM 43111 = ATCC 23218 = JCM 7437) is the type strain of *Nocardiopsis dassonvillei,* which in turn is the type species of the genus *Nocardiopsis*. Currently, *N. dassonvillei* is one of 40 validly published species belonging to the genus. The genus name derives from the Greek name *opsis,* appearance, and from Edmond Nocard, who first described in 1888 the type species of the genus *Nocardia*, *N. farcinica* [1,2]. *Nocardiopsis* means “that which has the appearance of *Nocardia*”. The species epithet is chosen in honor of Charles Dassonville, a contemporary French veterinarian [3]. The genus *Nocardiopsis* was first described by Meyer in 1976 [4] for bacteria that were previously classified as either *Streptothrix dassonvillei* (Brocq-Rousseau 1904) [3], *Nocardia dassonvillei* [5], or *Actinomadura dassonvillei* [6] on the basis of their morphological characteristics and cell wall type [4]. The strain IMRU 509^T^ is the neotype of the species *N. dassonvillei* (Brocq-Rousseau 1904). Databases provide contradictory speculations on the ecological and geographical origin of strain IMRU 509^T^ (e.g., soil from Paris, France; mildewed grain of unspecified geographical origin), however, solid information could not be extracted from the original literature [4,5,7-9]. Members of this species can be isolated from a variety of different habitats, including mildewed grain and fodder [3], different soils [10-13], antartic glacier [14], marine sediments [10,15], actinoryzal plant rhizosphere [16], gut tract of animals [17], active stalactites [18], cotton waste and occasionally in hay [19], air of a cattle barn [20], atmosphere of a composting facility [21], salterns [22] and from patients suffering from conjunctivitis [23] or cholangitis [8]. *N. dassonvillei* strains were also isolated from nodules and draining sinuses associate with an actinomycetoma of the anterior aspect of the right leg below the knee of a 39-year-old man [24]. A microorganism identical to *Streptothrix dassonvillei* was isolated two years later, but was placed in the genus *Nocardia* and designated *N. dassonvillei* [23]. Subsequently, the genus *Actinomadura* was described to harbor, among other species, also *N. dassonvillei* (Brocq-Rousseau) Liegard and Landrieu [4,8]. Further analysis supplied evidence that *A. dassonvillei* is not related to nocardiae [7]. Therefore, a new genus was created for *A. dassonvillei* on the basis of the characteristic development of spores, including the specific zig-zag formation of aerial hyphae before spore dispersal and the lack of madurose [4]. In 1976, *A. dassonvillei* was transferred to this new genus and was designated *Nocardiopsis dassonvillei* [4]. Also, *N. dassonvillei* is an earlier heterotypic synonym of *N. alborubida* [25]. The species epithet *alborubida* was considered as orthographically incorrect and corrected by Evtushenko to *albirubida* [10]. Subsequently, the species *N. dassonvillei* has been divided into three subspecies, namely subsp. *prasina* [26], subsp. *albirubida* (Grund and Kroppenstedt 1990) [10] and subsp. *dassonvillei* (Brocq-Rousseau 1904) [4,27], which is an earlier heterotypic synonym of *Streptomyces flavidofuscus* Preobrazhenskaya 1986 [28]. DNA-DNA hybridization data, as well as the results of biochemical tests, indicated that *N. alborubida* DSM 40465, *N. antarctica* DSM 43884, and *N. dassonvillei* DSM 43111 represent a single species designated *N. dassonvillei* [25]. Here we present a summary classification and a set of features for *N. dassonvillei* strain IMRU 509^T^, together with the description of the complete genomic sequencing and annotation.

## Classification and features

The 16S rRNA gene sequences of the strain IMRU 509^T^ share 95.9 to 99.5% sequence similarity with the 16S rRNA gene sequences of the type strains from the other members of the genus *Nocardiopsis* [29] The 16S rRNA gene of the strain IMRU 509^T^ also shares 99% similarity with an uncultured 16S rRNA gene sequence of the clone AKIW919 from urban aerosol in USA [30], but none of the sequences in metagenomic libraries (env_nt) shares more than 89% sequence identity, indicating that members of the species, genus and even family are poorly represented in the habitats screened thus far (as of November 2010). A representative genomic 16S rRNA sequence of *N. dassonvillei* was compared with the most recent release of the Greengenes database [31] using NCBI BLAST under default values and the relative frequencies of taxa and keywords, weighted by BLAST scores, were determined. The three most frequent genera were *Nocardiopsis* (91.1%), *Streptomyces* (7.1%) and *Prauseria* (1.8%). The species yielding the highest score was *N. dassonvillei* (including hits to *N. dassonvillei* subsp. *dassonvillei,* formerly also known as *Streptomyces flavidofuscus* [9,28]). The five most frequent keywords within the labels of environmental samples which yielded hits were 'soil(s)' (15.4%), ‘algeria, nocardiopsis, saccharothrix, saharan' (5.7%), 'source' (2.0%) and 'alkaline' (2.0%). These keywords fit to the morphology of the type strain as well as to the ecology of habitats from which the type strain and also other members of the species were isolated. The single most frequent keyword within the labels of environmental samples which yielded hits of a higher score than the highest scoring species was 'desert/soil' (50.0%).

**Figure 1:**
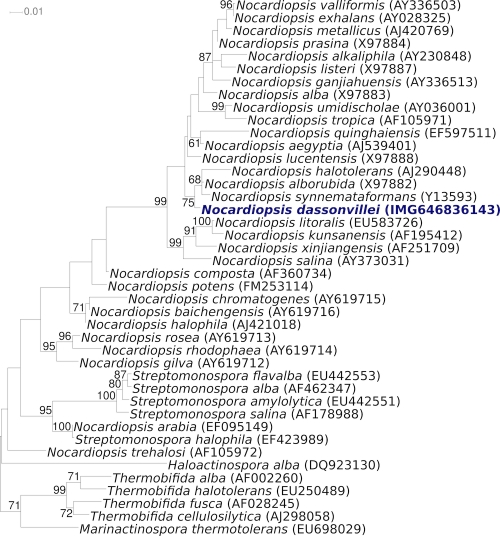
Phylogenetic tree highlighting the position of *N. dassonvillei* strain IMRU 509^T^ relative to the type strains of the other species within the genus and to the type strains of the other genera within the family *Nocardiopsaceae*. The trees were inferred from 1,442 aligned characters [32,33] of the 16S rRNA gene sequence under the maximum likelihood criterion [34] and rooted in accordance with the current taxonomy [35]. The branches are scaled in terms of the expected number of substitutions per site. Numbers above branches are support values from 750 bootstrap replicates [36] if larger than 60%. Lineages with type strain genome sequencing projects registered in GOLD [37] are shown in blue, published genomes in bold [38]. Note that the tree is more in accordance with the view of Grund and Kroppenstedt (1990) [39] to treat *N. alborubida* as a species of its own, rather than with the view of Yassin *et al*. (1997) [25] and Evtushenko *et al*. 2000 [10] to regard it as a subspecies of *N. dassonvillei* based on a 71% DDH value [10].

Figure 1 shows the phylogenetic neighborhood of *N. dassonvillei* strain IMRU 509^T^ in a 16S rRNA based tree. The sequences of the five 16S rRNA gene copies in the genome differ from each other by up to ten nucleotides, and differ by up to eight nucleotides from the previously published 16S rRNA sequence (X97886).

**Figure 2:**
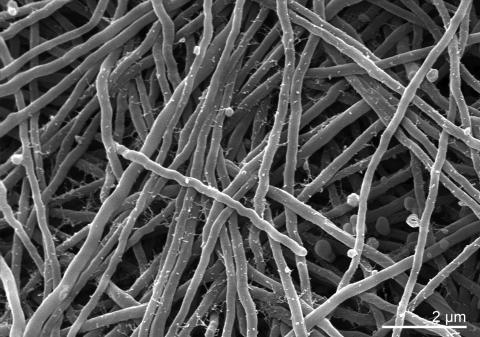
Scanning electron micrograph of *N. dassonvillei* strain IMRU 509^T^

The cells of strain IMRU 509^T^ are aerobic and Gram-positive [4]. (Table 1). Aerial mycelia are long, moderately branched, and, at the beginning of sporulation, more or less zig-zag-shaped (Figure 2). Later, the hyphae are straight or somewhat coiled [4]. They then divide into long segments which subsequently subdivide into smaller spores of irregular size [4]. Spores are elongated and smooth. Depending on the medium used, the color of the substrate mycelium is either yellowish-brown or olive to dark brown [4]. The aerial mycelium varies from a sparse coating to a thick, farinaceous to woolly cover of the colonies on oatmeal agar, oatmeal-nitrate agar, Bennett agar, Czapek-sucrose agar, inorganic salt-starch agar, yeast extract-malt extract agar, and complex organic medium 79 [4] of Prauser and Falta [51]. The color of the aerial mycelium is white or yellowish to grayish [4]. Colonies of substrate mycelia have dense filamentous margins [4]. Hyphae of the substrate mycelium fragment into coccoid elements after 3 to 4 weeks, depending on the medium used [4]. Soluble pigment is not produced [4]. Melanoid pigments are not produced on ISP 6 or tyrosine agar [4]. Growth of strain IMRU 509^T^ was tested on basal medium with and without carbohydrates. No growth was detected in the absence of carbohydrates. Strain IMRU 509^T^ was able to use N-acetyl-D-glucosamine, *p*-arbutin, D-galactose, gluconate, D-maltose, D-ribose, salicin, D-threalose, maltitol, putrescine, 4-aminobutyrate, azelate, citrate, fumarate, DL-lactate,L-alanine, β-alanine, L-aspartate, L-leucine and phenylacetate as sole carbon sources, but not *α*-D-melibiose, acetate, propionate, glutarate, L-malate, mesaconate, oxoglutarate, pyruvate, suberate, L-histidine, L-phenylalanine, L-proline, L-serine, L-tryptophan and 4-hydroxybenzoate [52]. However, L- arabinose, D-xylose, D-mannose, D-glucose, L-rhamnose, maltose, D-mannitol, D-fructose, sucrose and glycerol are the main carbohydrates used [4]. Acid is produced from L-arabinose, galactose, mannitol, sucrose and D-xylose [8]. Moreover, adonitol, dulcitol, *i*-inositol are not utilized [4]. L-alanine, proline and serine are also used as sole carbon as well as nitrogen sources, although proline and serine are weakly utilized [25]. Strain IMRU 509^T^ was found to hydrolyze p-nitrophenyl α-D-glucopyranoside, p-nitrophenyl β-D-glucopyranoside, p-nitrophenyl phenylphosphonate and L-alanine p-nitroanilide, but not aesculin, bis-p-nitrophenyl phosphate, p-nitrophenyl phosphorylcholine, L-glutamate-γ-3-carboxy-p-nitroanilide and L-proline p-nitroanilide [52]. Meyer (1976) reported that IMRU 509^T^ was not able to liquefy gelatin [4], while Yassin *et al*. reported in 1997 the opposite [25]. Strain IMRU 509^T^ is able to hydrolyze starch, to peptonize milk, to decompose esculin and to reduce nitrate to nitrite [4]. Strains of *N. dassonvillei* show positive tests of the decarboxylation of lactate, oxalate and propionate [8]. They also decompose casein, tyrosine and Tween 85. They show optimal growth at mildly alkaline conditions of pH 8, and at a salinity of 0% NaCl [8]. No growth is observed at 20% NaCl or at 45°C [8]. The catalase test is positive [4]. Strain IMRU 509^T^ hydrolyses adenine, xanthine and hypoxanthine [25]. 

**Table 1:** Classification and general features of *N. dassonvillei* strain IMRU 509^T^ according to the MIGS recommendations [40]

**MIGS ID**	**Property**	**Term**	**Evidence code**
	Current classification	Domain *Bacteria*	TAS [41]
		Phylum *Actinobacteria*	TAS [42]
		Class *Actinobacteria*	TAS [43]
		Order *Actinomycetales*	TAS [43-46]
		Family *Nocardiopsaceae*	TAS [9,46,47]
		Genus *Nocardiopsis*	TAS [4,48]
		Species *Nocardiopsis dassonvillei*	TAS [4,48]
		Type strain IMRU 509	TAS [4]
	Gram stain	positive	TAS [4]
	Cell shape	zig-zag shaped mycelium	NAS [4]
	Motility	none	NAS
	Sporulation	yes	TAS [4]
	Temperature range	mesophile, up to 42°C, but not 45°C	TAS [9]
	Optimum temperature	about 28°C	IDA
	Salinity	probably 0% NaCl, no growth at 20% NaCl	NAS
MIGS-22	Oxygen requirement	aerobic	TAS [4]
	Carbon source	carbohydrates	TAS [4]
	Energy source	L-arabinose, D-xylose, D-mannose, D-glucose, L-rhamnose, maltose, lactose, adonitol, dulcitol, D-mannitol, i-inositol, D-fructose, sucrose, raffinose, and glycerol.	TAS [4]
MIGS-6	Habitat	soil, mildewed grain, and clinical materials of animal and human origin	TAS [4]
MIGS-15	Biotic relationship	free-living	NAS
MIGS-14	Pathogenicity	actinomycetoma, conjunctivitis, cholangitis	TAS [8,23,24]
	Biosafety level	2	TAS [49]
	Isolation	not clearly reported	NAS
MIGS-4	Geographic location	probably Paris, France	NAS
MIGS-5	Sample collection time	not reported	TAS
MIGS-4.1 MIGS-4.2	Latitude Longitude	48.85 2.35 or other (see text)	NAS
MIGS-4.3	Depth	not reported	NAS
MIGS-4.4	Altitude	not reported	NAS

Evidence codes - IDA: Inferred from Direct Assay (first time in publication); TAS: Traceable Author Statement (i.e., a direct report exists in the literature); NAS: Non-traceable Author Statement (i.e., not directly observed for the living, isolated sample, but based on a generally accepted property for the species, or anecdotal evidence). These evidence codes are from of the Gene Ontology project [50]. If the evidence code is IDA, then the property was directly observed by one of the authors or an expert mentioned in the acknowledgements.

### Chemotaxonomy

The cell wall of the strain IMRU 509^T^ belongs to the chemotype III, which corresponds to the peptidoglycan type A1γ [53], i.e., N-acetyl-muramic acid, N-acetyl-glucosamine, alanine, glutamic acid, and *meso*-2, 6-diaminopimelic acid [4,8]. The products of the degradation of the cell wall are glycerol and glucose [8]. Strain IMRU 509^T^ is susceptible to lysozyme [4]. The polar lipids found in strain IMRU 509^T^ are phosphatidylinositol mannosides (PIM), phosphatidylinositol (PI), phosphatidylcholine (PC), monomannosyl diglyceride (MDG), phosphatidylglycerol (PG), phosphatidylmethylethanolamine (PME), monoacetylated glucose (AG), diphosphatidyl-glycerol (DPG), unknown phospholipids specific for *Nocardiopsis*, β-lipids of unknown structure (PL) [8]. The menaquinone type 4C2 was detected [8]. The menaquinone patterns of the strain IMRU 509^T^ contain menaquinones from MK-10 to MK-10(H_8_) and sugar type C [8]. Small amounts of the MK-9 and /or MK-12 series are also found [8]. The main fatty acids detected in the strain IMRU509^T^ were, *iso*-C_16:0_ (26.7%), *anteiso*-C_17:0_ (19.8%) and C_18:1_ (18.3%). Minor fatty acids detected included C_18:0_ (5.8%), C_17:1_ (5.2%), *anteiso*-C_15:0_ (3.2%), C_16:0_ (2.2%), *iso*-C_17:0_ (2.1%), C_16:1_ (1.2%) and *iso*-C_15:0_ (0.8%) [10].

## Genome sequencing and annotation

### Genome project history

This organism was selected for sequencing on the basis of its phylogenetic position [54], and is part of the *** G****enomic* *** E****ncyclopedia of* *** B****acteria and* *** A****rchaea * project [55]. The genome project is deposited in the Genome OnLine Database [37] and the complete genome sequence is deposited in GenBank. Sequencing, finishing and annotation were performed by the DOE Joint Genome Institute (JGI). A summary of the project information is shown in Table 2.

**Table 2:** Genome sequencing project information

**MIGS ID**	**Property**	**Term**
MIGS-31	Finishing quality	Finished
MIGS-28	Libraries used	Genomic libraries: one Sanger 8 kb pMCL200 library, one fosmid (pcc1Fos) library, one 454 pyrosequence standard library and one Illumina standard library
MIGS-29	Sequencing platforms	ABI3730, 454 GS FLX Titanium, Illumina GAii
MIGS-31.2	Sequencing coverage	9.0 × Sanger; 19.8 × pyrosequence; 22.3 × Illumina
MIGS-30	Assemblers	Newbler version 1.1.03.24, phrap
MIGS-32	Gene calling method	Prodigal 1.4, GenePRIMP
	INSDC ID	CP002040 chromosome CP002041 plasmid
	Genbank Date of Release	June 4, 2010
	GOLD ID	Gc01339
	NCBI project ID	19709
	Database: IMG-GEBA	646564557
MIGS-13	Source material identifier	DSM 43111
	Project relevance	Tree of Life, GEBA

### Growth conditions and DNA isolation

*N. dassonvillei* strain IMRU 509^T^, DSM 43111, was grown in DSMZ medium 65 (GYM *Streptomyces* medium) [56] at 28°C. DNA was isolated from 0.5-1 g of cell paste using Qiagen Genomic 500 DNA Kit (Qiagen, Hilden, Germany) following the standard protocol as recommended by the manufacturer, with modification st/DALM for cell lysis as described in Wu *et al*. [55].

### Genome sequencing and assembly

The genome was sequenced using a combination of Sanger and 454 sequencing platforms. All general aspects of library construction and sequencing can be found at the JGI website [57]. Pyrosequencing reads were assembled using the Newbler assembler version 2.1-PreRelease (Roche). Large Newbler contigs were broken into 6,356 overlap ping fragments of 1,000 bp and entered into assembly as pseudo-reads. The sequences were assigned quality scores based on Newbler consensus q-scores with modifications to account for overlap redundancy and adjust inflated q-scores. A hybrid 454/Sanger assembly was made using the PGA assembler. Possible mis-assemblies were corrected and gaps between contigs were closed by by editing in Consed, by custom primer walks from sub-clones or PCR products. A total of 462 Sanger finishing reads were produced to close gaps, to resolve repetitive regions, and to raise the quality of the finished sequence. Illumina reads were used to improve the final consensus quality using an in-house developed tool (the Polisher ) [58]. The error rate of the completed genome sequence is less than 1 in 100,000. Together, the combination of the Sanger and 454 sequencing platforms provided 28.77 × coverage of the genome. The final assembly contains 68,385 Sanger reads and 1,376,163 pyrosequencing reads.

### Genome annotation

Genes were identified using Prodigal [59] as part of the Oak Ridge National Laboratory genome annotation pipeline, followed by a round of manual curation using the JGI GenePRIMP pipeline [60]. The predicted CDSs were translated and used to search the National Center for Biotechnology Information (NCBI) nonredundant database, UniProt, TIGRFam, Pfam, PRIAM, KEGG, COG, and InterPro databases. Additional gene prediction analysis and functional annotation was performed within the Integrated Microbial Genomes - Expert Review (IMG-ER) platform [61].

## Genome properties

The genome consists of a 5,767,958 bp long chromosome with a 73% GC content, and a 775,354 bp long plasmid a 72% GC content (Table 3 and Figure 3a and Figure 3b). Of the 5,647 genes predicted, 5,570 were protein-coding genes, and 77 RNAs; 73 pseudogenes were also identified. The majority of the protein-coding genes (69.6%) were assigned with a putative function while the remaining ones were annotated as hypothetical proteins. The distribution of genes into COGs functional categories is presented in Table 4.

**Table 3:** Genome Statistics

**Attribute**	**Value**	**% of Total**
Genome size (bp)	6,543,312	100.00%
DNA coding region (bp)	5,543,886	84.73%
DNA G+C content (bp)	4,758,935	72.73%
Number of replicons	2	
Extrachromosomal elements	1	
Total genes	5,647	100.00%
RNA genes	77	1.36%
rRNA operons	5	
Protein-coding genes	5,570	98.64%
Pseudo genes	73	1.29%2
Genes with function prediction	3,930	69.59%
Genes in paralog clusters	1,055	18.68%
Genes assigned to COGs	3,793	67.17%
Genes assigned Pfam domains	4,204	74.45%
Genes with signal peptides	1,686	29.86%
Genes with transmembrane helices	1,337	23.68%
CRISPR repeats	8	

**Figure 3a:**
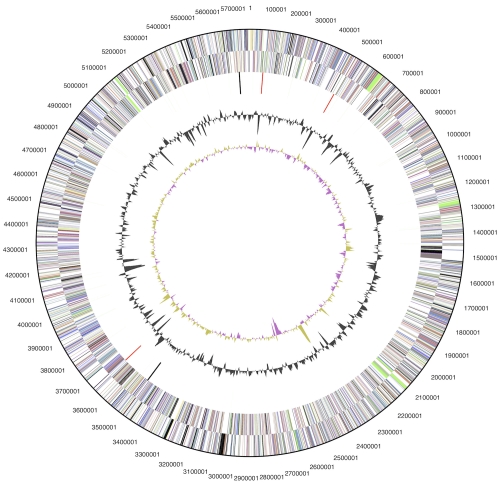
Graphical circular map of the chromosome (not drawn to scale with plasmid). From outside to the center: Genes on forward strand (color by COG categories), Genes on reverse strand (color by COG categories), RNA genes (tRNAs green, rRNAs red, other RNAs black), GC content, GC skew.

**Figure 3b:**
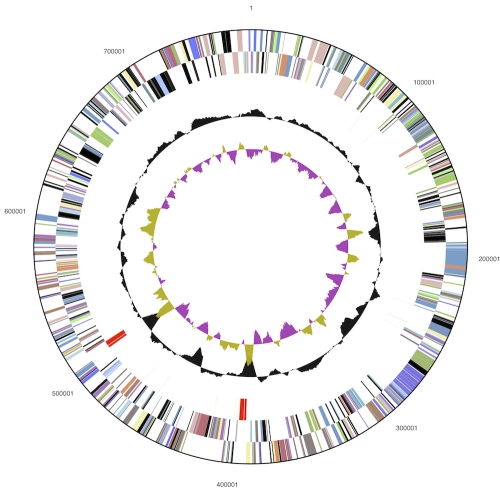
Graphical circular map of the plasmid (not drawn to scale with chromosome). From outside to the center: Genes on forward strand (color by COG categories), Genes on reverse strand (color by COG categories), RNA genes (tRNAs green, rRNAs red, other RNAs black), GC content, GC skew.

**Table 4:** Number of genes associated with the general COG functional categories

Code	value	%age	Description
J	180	4.1	Translation, ribosomal structure and biogenesis
A	1	0.0	RNA processing and modification
K	518	11.9	Transcription
L	173	4.0	Replication, recombination and repair
B	1	0.0	Chromatin structure and dynamics
D	38	0.9	Cell cycle control, cell division, chromosome partitioning
Y	0	0.0	Nuclear structure
V	103	2.4	Defense mechanisms
T	279	6.4	Signal transduction mechanisms
M	190	4.4	Cell wall/membrane/envelope biogenesis
N	3	0.1	Cell motility
Z	2	0.1	Cytoskeleton
W	0	0.0	Extracellular structures
U	35	0.8	Intracellular trafficking and secretion, and vesicular transport
O	115	2.6	Posttranslational modification, protein turnover, chaperones
C	266	6.1	Energy production and conversion
G	371	8.5	Carbohydrate transport and metabolism
E	354	8.1	Amino acid transport and metabolism
F	102	2.3	Nucleotide transport and metabolism
H	210	4.8	Coenzyme transport and metabolism
I	184	4.2	Lipid transport and metabolism
P	212	4.9	Inorganic ion transport and metabolism
Q	144	3.3	Secondary metabolites biosynthesis, transport and catabolism
R	578	13.3	General function prediction only
S	295	6. 8	Function unknown
-	1,854	32.8	Not in COGs
